# Vitamin D Analogs Differentially Control Antimicrobial Peptide/“Alarmin”Expression in Psoriasis

**DOI:** 10.1371/journal.pone.0006340

**Published:** 2009-07-22

**Authors:** Mark Peric, Sarah Koglin, Yvonne Dombrowski, Katrin Groß, Eva Bradac, Amanda Büchau, Andreas Steinmeyer, Ulrich Zügel, Thomas Ruzicka, Jürgen Schauber

**Affiliations:** 1 Department of Dermatology and Allergology, Ludwig-Maximilians-University, Munich, Germany; 2 Common Mechanism Research Early Projects, Global Drug Discovery, Bayer Schering Pharma AG, Berlin, Germany; 3 Medicinal Chemistry, Global Drug Discovery, Bayer Schering Pharma AG, Berlin, Germany; New York University School of Medicine, United States of America

## Abstract

Antimicrobial peptides (AMPs) are strongly expressed in lesional skin in psoriasis and play an important role as proinflammatory “alarmins” in this chronic skin disease. Vitamin D analogs like calcipotriol have antipsoriatic effects and might mediate this effect by changing AMP expression. In this study, keratinocytes in lesional psoriatic plaques showed decreased expression of the AMPs β-defensin (HBD) 2 and HBD3 after topical treatment with calcipotriol. At the same time, calcipotriol normalized the proinflammatory cytokine milieu and decreased interleukin (IL)-17A, IL-17F and IL-8 transcript abundance in lesional psoriatic skin. In contrast, cathelicidin antimicrobial peptide expression was increased by calcipotriol while psoriasin expression remained unchanged. In cultured human epidermal keratinocytes the effect of different vitamin D analogs on the expression of AMPs was further analyzed. All vitamin D analogs tested blocked IL-17A induced HBD2 expression by increasing IκB-α protein and inhibition of NF-κB signaling. At the same time vitamin D analogs induced cathelicidin through activation of the vitamin D receptor and MEK/ERK signaling. These studies suggest that vitamin D analogs differentially alter AMP expression in lesional psoriatic skin and cultured keratinocytes. Balancing AMP “alarmin” expression might be a novel goal in treatment of chronic inflammatory skin diseases.

## Introduction

Psoriasis is a chronic inflammatory skin disorder which affects approximately 2% of the general population. Although the specific cause for psoriasis is unknown there is strong evidence for a genetic basis of the disease [1], [2]. Furthermore, a large body of evidence has identified a dysregulated interplay between keratinocytes and infiltrating immune cells underlying cutaneous inflammation in psoriasis [3]. Cytokines and other soluble factors such as antimicrobial peptides (AMPs) secreted by resident or infiltrating cells are essential elements in this process of cell-cell communication. Initially, AMPs were characterized as effector molecules of innate immunity as they provide a first barrier of defence against microbial pathogens [4]. In the meantime, an array of additional functions of AMPs has been identified and due to their multiple functions as activators of adaptive immune responses and inflammation the term “alarmins” has been introduced [5]. Two families of AMPs are among the best characterized for their “alarmin” function: the defensins and the cathelicidins.

Recent publications highlight the role of dysregulated expression of AMPs in the pathogenesis of psoriasis. Human β-defensins (HBD) and cathelicidin [6], as well as psoriasin and other AMPs [7]–[9], are strongly increased in keratinocytes in psoriatic plaques. In addition, Hollox et al. were able to show that higher genomic copy numbers of β-defensin genes correlate with an increased risk to develop psoriasis [1]. Recently it was demonstrated that cathelicidin peptide LL-37 is able to suppress apoptosis induction in keratinocytes [10]. Deregulation of apoptosis control is characteristic for keratinocytes in psoriasis and increased cathelicidin in psoriatic plaques could explain this clinical phenotype. Furthermore, a mechanism was described by which plasmacytoid dendritic cells (pDCs) sense and respond to self-DNA coupled with the cathelicidin LL-37, thereby driving autoimmunity in psoriasis [11]. Thus, as AMPs might play a major role in the pathogenesis of psoriasis and skin inflammation controlling AMP expression might offer a novel approach to psoriasis treatment.

The mechanisms of AMP regulation in keratinocytes in psoriasis are incompletely understood. Recent studies have highlighted a role for the Th17 cytokines interleukin- (IL-) 17 and IL-22 in mediating cutaneous skin inflammation in psoriasis [12], [13]. Additionally, serum levels of IL-8 and IL-17 were shown to correlate with severity of the cutaneous disease in psoriasis patients [14], [15]. HBD2 and HBD3 were shown to be regulated by IL-22 via STAT3 activation [16], [17] and by IL-17A through JAK and NF-κB [18], [19]. In keratinocytes, human cathelicidin is directly regulated by 1,25-dihydroxyvitamin D3 (1,25D3) through a vitamin D3 responsive element (VDRE) in the 5′ untranslated region (UTR) of its gene CAMP [20]. Interestingly, cytokines and other mediators of skin inflammation do not induce cathelicidin in keratinocytes *in vitro* [21]. However, in the presence of 1,25D3 human keratinocytes upregulate cathelicidin expression in response to toll-like receptor (TLR) 2 and IL-17 signaling [22], [23].

These observations point out a paradox in the understanding of the role of cathelicidin in psoriasis pathogenesis: vitamin D analogs like calcipotriol are very effective in normalizing the skin phenotype when applied topically to psoriatic skin [24], [25]. As vitamin D analogs activate the vitamin D receptor (VDR) in keratinocytes one would expect that they also induce cathelicidin. Increased cathelicidin “alarmin” should then aggravate inflammation by binding self-DNA and activating pDCs. Still, the opposite is true: vitamin D analogs are a mainstay in the topical treatment of psoriasis.

To date there are no published studies that have assessed the effect of vitamin D analogs on AMP expression in keratinocytes in psoriasis. In this trial we analyzed the expression of several AMPs including β-defensins and cathelicidin in psoriatic plaques before and after treatment with the vitamin D analog calcipotriol. Treatment with calcipotriol led to clinical improvement and a decrease in inflammatory parameters such as IL-17A, IL-17F and IL-8 in psoriatic skin. In parallel, expression levels of HBD2 and HBD3 decreased. In contrast, cathelicidin was induced in psoriatic skin after treatment with calcipotriol. These observations were confirmed *in vitro*: various vitamin D analogs decreased inflammatory responses in human keratinocytes and reduced β-defensin expression through inhibition of Nf-κB activation. In contrast, all vitamin D analogs tested induced cathelicidin hCAP-18 through activation of the vitamin D receptor and a modest involvement of MEK/ERK signaling.

## Results

### Changes in antimicrobial peptide and inflammatory cytokine expression in psoriatic plaques after treatment with calcipotriol

**Figure 1:**
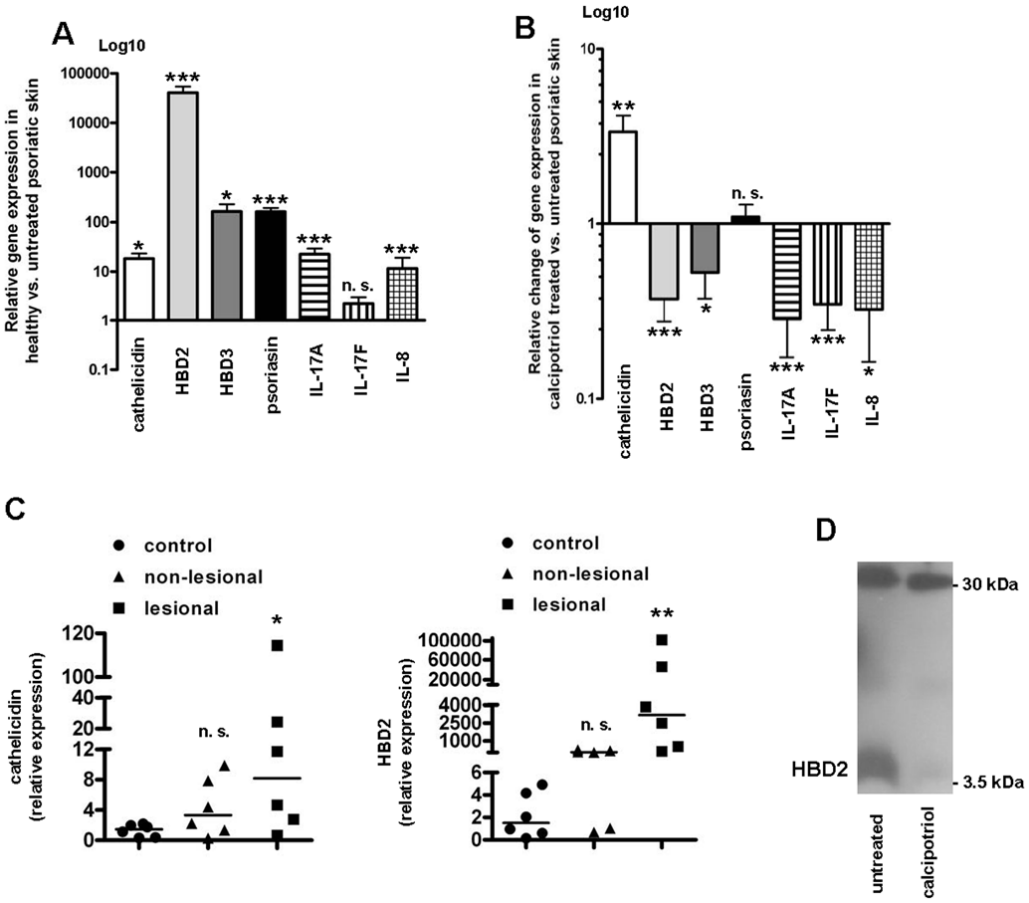
Expression of antimicrobial peptides in psoriatic plaques before and after treatment with vitamin D analogs. 4-mm punch biopsies from a marker psoriatic plaque were taken from patients (n = 8) before and after treatment with ointment containing the vitamin D analog calcipotriol (0.005%; applied twice daily for 5 to 7 days). Total mRNA was extracted and transcript levels of cathelicidin, HBD2, HBD3, psoriasin, IL-17A, IL-17F and IL-8 were analyzed by qPCR. Expression of antimicrobial peptides and markers of inflammation in untreated, lesional skin of psoriasis patients was normalized to the mean expression of target genes in skin of the healthy (non-psoriatic) controls (n = 7) (A). In (B) the relative changes of gene expression levels in calcipotriol treated vs. untreated lesional skin of psoriasis patients are displayed. The Y-axis in (A) and (B) is depicted in Log10 scale (n. s. not significant, *P<0.05, **P<0.01, *** *P*<0.001; Mann-Whitney test). (C) Punch biopsies from lesional and non-lesional psoriatic plaque were taken from patients (n = 6). Total mRNA was extracted and transcript levels of cathelicidin (left panel) and HBD2 (right panel) analyzed by qPCR. Statistical analysis of lesional or non-lesional, respectively, psoriatic skin vs. healthy (non-psoriatic) controls (n = 6) was performed with Mann-Whitney test, comparison of lesional vs. non-lesional biopsies was performed with Wilcoxon matched pairs test (n. s. not significant, *P<0.05, **P<0.01). In (D) Western blot analysis using an antibody which detects HBD2 was performed with total protein extracted from biopsies taken from lesional skin of one representative psoriasis patient before and after treatment with calcipotriol.

The vitamin D analog calcipotriol is widely and successfully used in the topical treatment of psoriasis [26]. Cutaneous inflammation is usually normalized within a few days after the onset of therapy. Nevertheless, the mechanisms and signaling pathways involved in the effects of calcipotriol are not completely elucidated. The expression of various AMPs such as cathelicidin hCAP18/LL-37 and β-defensins is increased in psoriatic plaques [7], [27]. Beyond their antimicrobial function AMPs can act as proinflammatory “alarmins” and recent data link these peptides directly to psoriasis pathogenesis. Indeed, expression of cathelicidin, HBD2, HBD3 and psoriasin were significantly increased in psoriatic plaques (Figure 1A). At the same time markers of inflammation such as IL-17A, IL-17F and IL-8 were elevated confirming earlier results [28]. Topical treatment with calcipotriol decreased cutaneous inflammation as demonstrated by decreased IL-17A, IL-17F and IL-8 and significantly lowered HBD2 and HBD3 transcript abundance (Figure 1B). In contrast, treatment with calcipotriol increased cathelicidin levels in psoriatic plaques whereas levels of psoriasin were unchanged (Figure 1B). Analysis of biopsies from lesional and non-lesional psoriatic skin confirmed significantly elevated levels of cathelicidin and HBD2 in lesional psoriatic skin compared to healthy skin (non-psoriatic) from control patients (Fig. 1C). Neither cathelicidin nor HBD2 mRNA expression was significantly elevated in non-lesional psoriatic skin vs. healthy (non-psoriatic) skin, but a trend towards increased expression was observed. The decrease in HBD2 peptide after calcipotriol treatment was confirmed as displayed in Figure 1D. Immunofluorescence stainings confirmed expression of HBD2 and cathelicidin in epidermal keratinocytes in psoriatic plaques before and after treatment (Figure S1).

### Vitamin D analogs block IL-17A induced HBD2 and IL-8 expression in human keratinocytes by inhibiting Nf-κB

**Figure 2:**
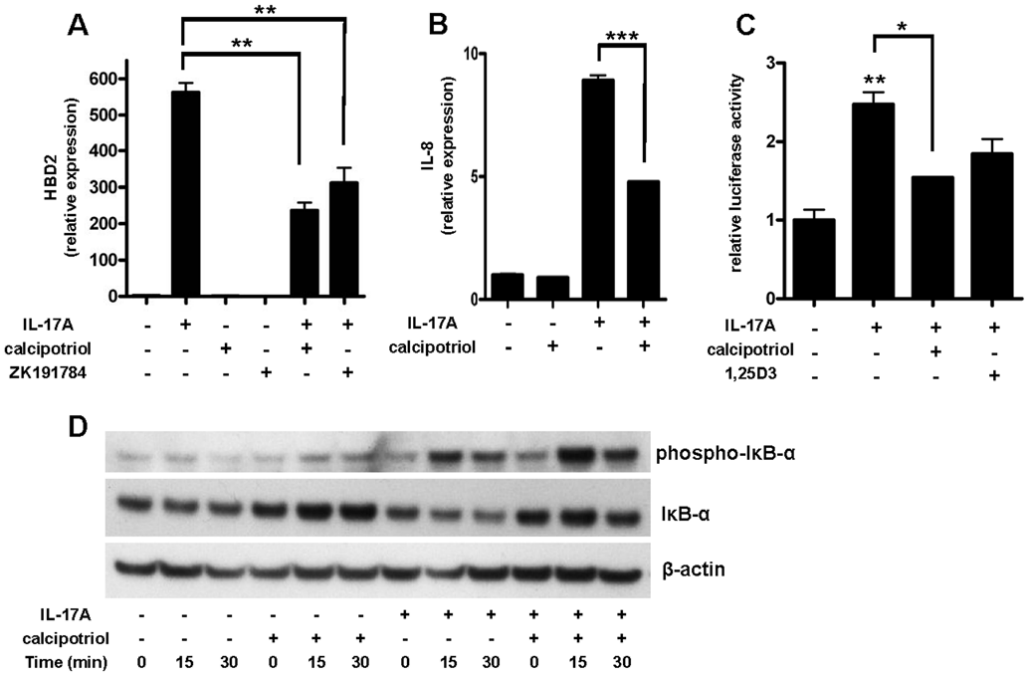
Induction of HBD2 and IL-8 by IL-17A is decreased by calcipotriol through inhibition of NF-κB signaling. (A) NHEK were stimulated with IL-17A (10 ng/ml) in the presence or absence of vitamin D analogs calcipotriol or ZK191784 (10^−8^ M). Cells were harvested after 24 h and HBD2 transcript levels were analyzed by qPCR. In (B) IL-8 transcript expression after stimulation of NHEK with IL-17A (10 ng/ml) in the presence or absence of calcipotriol (10^−8^ M) is displayed. Data are means±SD of a single experiment performed in triplicate and representative of 2 to 3 independent experiments (**P<0.01, ***P<0.001; Student's *t* test). (C) To study involvement of the Nf-κB pathway we performed reporter gene analyses with an Nf-κB reporter plasmid. 24 h after transfection of HaCaT keratinocytes cells were stimulated with IL-17A (10 ng/ml) in the presence or absence of calcipotriol or 1,25D3 (10^−8^ M). Luciferase activity was assayed (*P<0.05, **P<0.01; Student's *t* test). (D) To further confirm Nf-κB involvement cells were stimulated with IL-17A (10 ng/ml) in the presence or absence of calcipotriol (10^−8^ M) and harvested after 0, 15 or 30 min. Western blot analysis using antibodies against phospho-IκB-α and unphosphorylated IκB-α were performed to analyze activation of NF-κB. Staining for β-actin served as a loading control.

The understanding of the crucial role of IL-17A in skin inflammation in psoriasis is rapidly evolving [28]. Among its multiple activities IL-17A was recently identified as a very potent inducer of HBD2 [18]. HBD2 belongs to the β-defensin family and the β-defensins have been directly linked to psoriasis pathogenesis [1]. Therefore the effect of the vitamin D analogs calcipotriol and ZK191784 on IL-17A induced HBD2 expression was analyzed in keratinocytes *in vitro*. IL-17A strongly induced HBD2 transcript abundance in NHEK and induction was significantly reduced by calcipotriol and ZK191784 (Figure 2A). A dose-dependent inhibition of HBD2 induction was confirmed with increasing concentrations of calcipotriol (data not shown). At the same time the induction of IL-8 by IL-17A was decreased by calcipotriol (Figure 2B).

Next, we investigated the mechanism behind the antipsoriatic effect of the vitamin D analogs. HBD2 and IL-8 are both regulated by Nf-κB signaling in human epithelial cells [18], [19]. A negative regulator of Nf-κB activity is IκB-α which binds to NF-κB subunits in the cytoplasm preventing Nf-κB translocation to the nucleus and subsequent gene activation [29]. Upon activation of the Nf-κB pathway IκB-α is phosphorylated and subsequently degraded which results in the release of NF-κB subunits [29]. In additional analysis, the activity of NF-κB was significantly increased after stimulation with IL-17A in keratinocytes and this effect was reduced by co-stimulation with calcipotriol or 1,25D3, respectively (Figure 2C). In another approach, calcipotriol strongly increased the expression of IκB-α protein in keratinocytes (Figure 2D). As expected, stimulation with IL-17A resulted in higher levels of phosphorylated IκB-α and subsequent IκB-α degradation. When cells were stimulated with the combination of calcipotriol and IL-17A higher levels of phosphorylated IκB-α were observed (Figure 2D). Still, in contrast to stimulation with IL-17A alone, IκB-α was not degraded when cells were stimulated with calcipotriol and IL-17A which indicates an inhibition of Nf-κB activation by calcipotriol (Figure 2D).

### Vitamin D analogs induce cathelicidin expression in human keratinocytes

**Figure 3:**
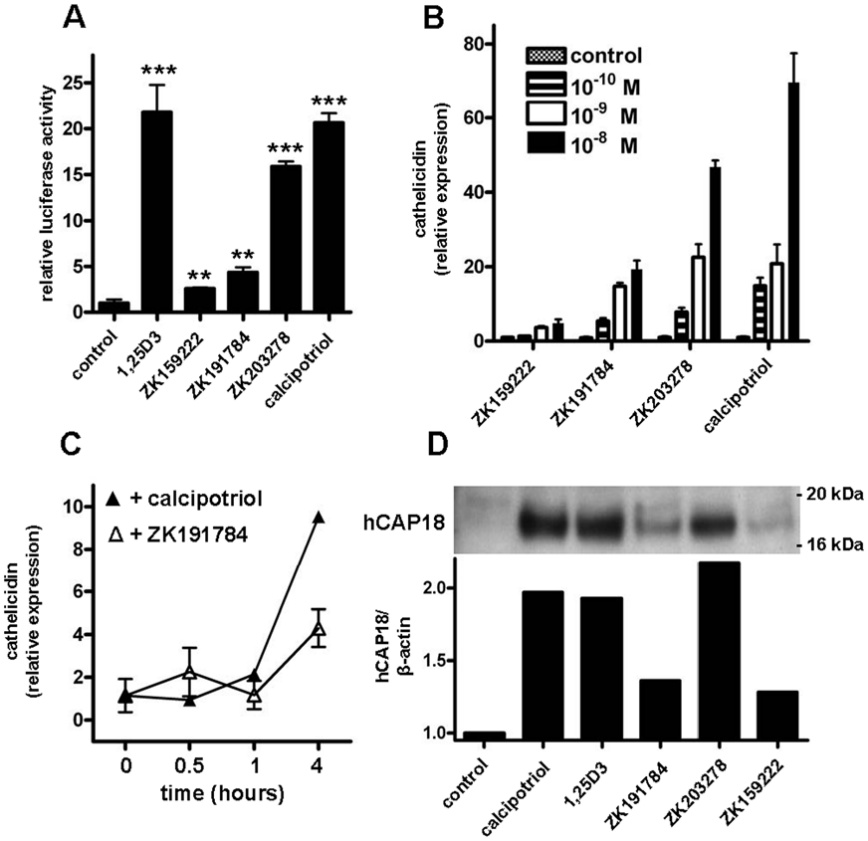
Vitamin D analogs enhance cathelicidin promoter activity and induce expression in primary human epidermal keratinocytes. (A) To analyze the effects of vitamin D analogs on the cathelicidin promoter a 5 kb fragment of the 5′ UTR of the cathelicidin gene CAMP was cloned into a luciferase reporter plasmid and transfected into HaCaT keratinocytes. Cells were stimulated with 1,25D3, ZK159222, ZK191784, ZK203278 and calcipotriol (all at 10^−7^ M) and luciferase activity was assayed (**P<0.01, ***P<0.001; Student's *t* test). In (B) primary human keratinocytes (NHEK) were stimulated with increasing concentrations of vitamin D analogs ZK159222, ZK191784, ZK203278 or calcipotriol (all: 10^−10^ M – 10^−8^ M). Cells were harvested after 24 h and cathelicidin transcript levels were analyzed by qPCR. (C) NHEK were stimulated with vitamin D analogs ZK191784 and calcipotriol (all at 10^−8^ M) for 0.5, 1 and 4 hours. Again, cathelicidin transcript abundance was analyzed by qPCR. All data are means±SD of a single experiment performed in triplicate and are representative of 2 to 3 independent experiments. (D) To evaluate cathelicidin peptide induction NHEK were treated with calcipotriol, 1,25D3, ZK191784, ZK203278 or ZK159222 (all at 10^−8^ M). Cathelicidin hCAP18 protein expression was analyzed in NHEK lysates by Western blot after 24 hours. Staining for β-actin served as loading control and hCAP18/β-actin ratios were analyzed using densitometry.

Cathelicidin is another antimicrobial peptide which acts as an “alarmin” in skin. In particular, cathelicidin peptide LL-37 has been identified as a critical factor for the activation of an inflammatory cascade in psoriasis [11]. Unexpectedly, despite clinical improvement and a decrease in inflammatory parameters after treatment of psoriasis plaques with calcipotriol the expression of cathelicidin was not decreased but increased (Figure 1B). To analyze the effect of vitamin D analogs on cathelicidin expression *in vitro* a 5 kb fragment of the 5′ untranslated region of the human cathelicidin gene CAMP was transfected in keratinocytes and transcriptional activity was assayed. All vitamin D analogs including calcipotriol enhanced cathelicidin promoter activity (Figure 3A). Dose- and time-dependent induction of cathelicidin transcript was confirmed in human primary keratinocytes (NHEK) (Figure 3B and C) and HaCaT keratinocytes (data not shown). When used at identical concentrations, calcipotriol exerted similar effects as 1,25D3, the effect of ZK203278 was slightly, the effect of ZK191784 significantly lower and ZK159222 showed the weakest induction of all analogs (Figure 3B). Simultaneously, all analogs tested increased the cathelicidin precursor protein hCAP18 in NHEK (Figure 3D). Processing of hCAP-18 to active LL-37 peptide could not be detected in our Western blot system.

### VDR and the MEK/ERK signaling pathway are involved in cathelicidin induction by vitamin D analogs

**Figure 4:**
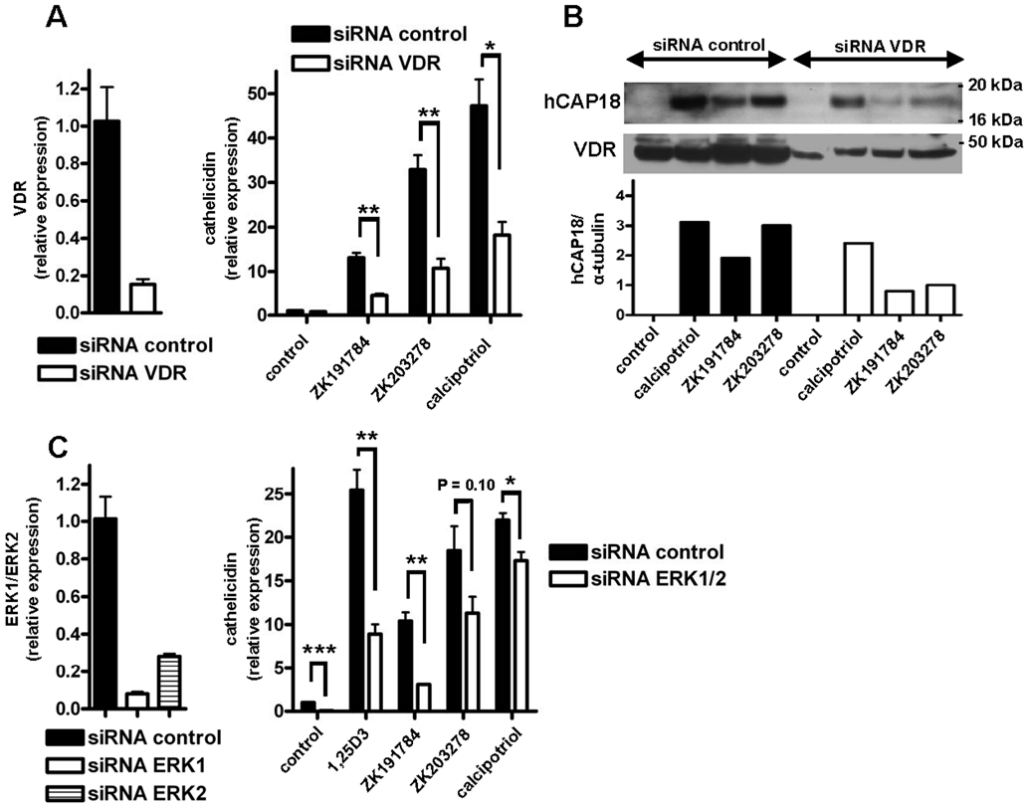
VDR and the MEK/ERK signaling pathway are involved in cathelicidin induction by vitamin D analogs. To characterize the role of the VDR in increased cathelicidin expression after treatment with vitamin D analogs, NHEK were transfected with siRNA to decrease VDR expression before stimulation with vitamin D analogs ZK191784, ZK203278 and calcipotriol (all at 10^−8^ M). Silencing of VDR was confirmed by qPCR (A; left panel) and Western blot (B; middle panel). siRNA suppression of VDR significantly reduced the induction of cathelicidin mRNA by all vitamin D analogs after 24 hours (A; right panel) (*P<0.05, **P<0.01; Student's *t* test). In (B; upper panel) the corresponding cathelicidin peptide hCAP18 expression levels are displayed as observed by Western blot. Staining for α-tubulin served as loading control and cathelicidin hCAP-18/α-tubulin ratios were analyzed using densitometry. (C) To analyze the role of MEK/ERK signaling in cathelicidin induction NHEK were transfected simultaneously with two different siRNAs (20 nM) to decrease ERK1 and ERK2 expression before stimulation with 1,25D3 and its analogs ZK191784, ZK203278 and calcipotriol (all at 10^−8^ M). Silencing of ERK1 and ERK2 was confirmed by qPCR (C; left panel). siRNA suppression of ERK1 and ERK2 resulted in reduced induction of cathelicidin mRNA by vitamin D analogs (C; right panel). Data are means±SD of a single experiment performed in triplicate and are representative of 2 to 3 independent experiments (*P<0.05, **P<0.01, ***P<0.001; Student's *t* test).

Having confirmed that vitamin D analogs induce cathelicidin expression underlying signaling pathways were investigated. 1,25D3 increases cathelicidin expression through activation of the vitamin D receptor [30]. To investigate if vitamin D analogs enhance cathelicidin also through the VDR siRNA experiments to silence VDR were performed. Silencing of the VDR (Figure 4A, left panel) significantly reduced the induction of cathelicidin transcript by ZK191784, ZK203278 or calcipotriol (Figure 4A, right panel). Similarly, Western blot analysis demonstrated silencing of VDR protein expression (Figure 4B, middle panel) which resulted in strongly reduced hCAP18 induction after stimulation with vitamin D analogs (Figure 4B, upper panel).

MEK/ERK signaling is critically involved in the regulation of cathelicidin by 1,25D3 in human keratinocytes [23]. To confirm involvement of MEK/ERK signaling in the induction of cathelicidin by vitamin D analogs, primary keratinocytes were transfected with a combination of siRNA oligonucleotides directed against ERK1 and ERK2 (Figure 4C, left panel). Compared to cells treated with control siRNA silencing of ERK1/ERK2 significantly reduced cathelicidin induction by 1,25D3 and its analogs ZK191784 and calcipotriol. Modest inhibition was observed for induction by ZK203278 (Figure 4C, right panel).

**Figure 5:**
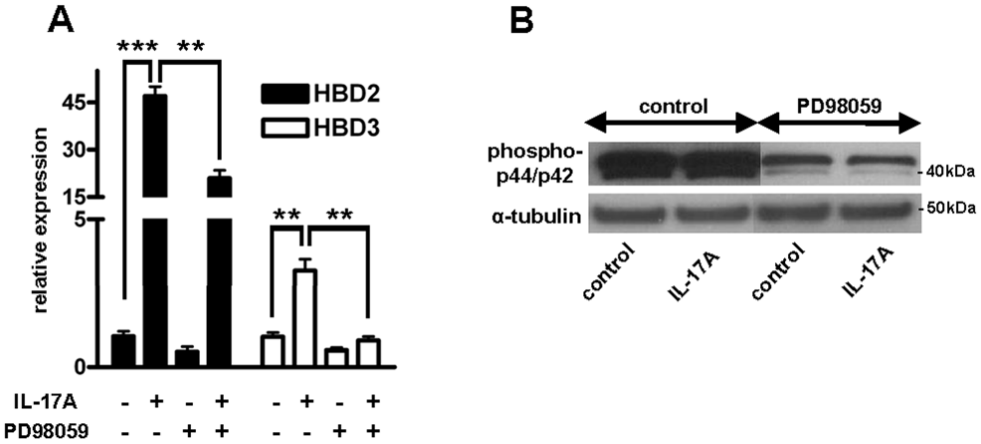
HBD2 and HBD3 expression is induced by IL-17A via a MEK/ERK-dependent mechanism. (A) To investigate the role of MEK/ERK signaling in HBD2 and HBD3 expression NHEK were treated with the specific MEK1 inhibitor PD98059 (20 µM) prior to stimulation with IL-17A (10 ng/ml). Cells were harvested after 24 h and transcript levels were analyzed by qPCR. Inhibition of MEK/ERK significantly blocked HBD2 and HBD3 induction by IL-17A. Data are means±SD of a single experiment performed in triplicate and are representative of 2 to 3 independent experiments (**P<0.01, ***P<0.001; Student's *t* test). The inhibitory effect of PD98059 on MEK/ERK was confirmed by Western blot analyses of phospho-p44/p42 (B, upper panel). Staining for α-tubulin served as loading control.

To analyze whether MEK/ERK signaling is also involved in β-defensin induction in keratinocytes additional experiments were performed. In parallel to cathelicidin induction, MEK/ERK inhibition by the specific MEK1 inhibitor PD98059 significantly diminished the effect of IL-17A on HBD2 and HBD3 (Figure 5A). Blocking of MEK/ERK by PD98059 was confirmed by Western blot using an antibody against phospho-p44/p42 (Figure 5B).

## Discussion

Vitamin D analogs are a hallmark in the treatment of psoriasis but the mechanisms behind their antipsoriatic actions are not completely understood. AMPs can act as proinflammatory mediators or “alarmins” and link adaptive and innate immune responses [4]. Recent studies suggest a role of the defensins and cathelicidin in the pathogenesis of skin inflammation in psoriasis [3]. In particular, increased gene copy numbers of the β-defensins (HBDs) correlate with the risk to develop this disease [1] and cathelicidin peptide, which is increased in psoriatic skin, induces an autoinflammatory cascade leading to skin inflammation [11]. As AMPs are directly involved in psoriasis pathogenesis targeting AMP expression or function might be a promising approach in the treatment of cutaneous inflammation in psoriasis.

In this study we demonstrate that treatment of lesional psoriatic skin with the vitamin D analog calcipotriol normalized the cutaneous phenotype and at the same time changes the AMP expression profile. Before treatment the levels of various AMPs were highly elevated in psoriatic plaques compared to healthy skin. Calcipotriol treatment effectively decreased epidermal proliferation but at the same time differentially affected AMP expression. While expression of HBD2 and HBD3 was strongly downregulated by calcipotriol the levels of cathelicidin were increased.

The impact of treatment on the expression of HBD2 peptide in inflamed skin in psoriatic plaques has only been studied in a few studies [31]. IL-17A, which is known to be a very potent inducer of HBD2 [18], is a central cytokine in psoriasis pathogenesis [32] and correlates with disease severity [14], [15]. In this study HBD2 and HBD3 were increased in untreated psoriatic plaques. At the same time markers of inflammation such as IL-17A, IL-17F and IL-8 were elevated. Topical treatment with calcipotriol significantly decreased cutaneous levels of HBD2 and HBD3 as well as IL-17A, IL-17F and IL-8.

To analyze the underlying mechanism primary human keratinocytes were stimulated with IL-17A which resulted in strong induction of HBD2 and IL-8. Both genes are regulated by Nf-κB signaling [18] and the presence of calcipotriol blocked HBD2 and IL-8 induction by inhibiting Nf-κB activation. In particular, the presence of calcipotriol increased the level of the Nf-κB inhibitor protein IκB-α in NHEK and inhibited IL-17A induced IκB-α degradation. This is in accordance with an earlier study in which Riis et al. showed that activation of the vitamin D signaling pathway increases IκB-α expression resulting in decreased NF-κB DNA binding activity to the IL-8 κB binding sequence [33]. Furthermore, vitamin D anaolgs inhibited NF-κB promoter activity in keratinocytes. Calcipotriol might therefore decrease HBD2 expression in keratinocytes by two mechanisms: by directly blocking the induction of HBD2 through inhibition of Nf-κB and by lowering the tissue levels of IL-17A. Decreased IL-17A expression might be due to decreased recruitment of circulating, proinflammatory T–cells to the skin which was observed earlier after treatment of inflamed skin with vitamin D analogs [34].

Similar to HBD2, cathelicidin antimicrobial peptide has been suggested to play an important role in psoriasis pathogenesis. Elevated levels of this AMP in psoriatic plaques have been published recently [6], [35] and autoinflammatory effects of the cathelicidin peptide LL-37 have been observed in inflamed skin in psoriasis [5], [11]. In addition, cathelicidin inhibits apoptosis in keratinocytes which could result in the altered phenotype of lesional psoriatic skin [10]. In the present study strongly elevated levels of cathelicidin in keratinocytes in psoriatic plaques were confirmed. Unexpectedly and despite clinical recovery, real-time PCR and immunofluorescence demonstrated increased cathelicidin expression in lesional skin compared to untreated skin after treatment with calcipotriol. At the same time IL-17F which is important for neutrophil recruitment was reduced indicating that the induction of cathelicidin was indeed due to increased keratinocytic expression and not to neutrophilic tissue infiltration [36].

This inducing effect of vitamin D analogs on cathelicidin expression was confirmed in cell culture experiments using primary keratinocytes: Vitamin D analogs such as calcipotriol induced cathelicidin through activation of the VDR and subsequent CAMP transcription. Interestingly, only cathelicidin precursor protein hCAP18 was increased after stimulation with vitamin D analogs but not active LL-37 peptide. In psoriatic plaques LL-37 binds self-DNA and activates pDCs to secrete IFN-α [11]. However, in our study IFN-α expression was not increased in psoriatic skin after treatment with calcipotriol (data not shown). Recently, it was also shown that vitamin D3 does not directly modulate IFN-α production in pDCs in human skin [37]. It might therefore be possible that the induction of hCAP18 is not accompanied by an increase of active LL-37 due to a lack of factors which are required for hCAP18 processing. Further studies are needed to identify the signaling events involved in controlled processing of cathelicidin peptides in skin.

Vitamin D signaling is crucial for the regulation of tissue homeostasis in the skin but also involved in the regulation of systemic calcium homeostasis. Treatment of hyperproliferative disorders such as psoriasis with vitamin D analogs might induce hypercalcemia even at a therapeutic dosage [38]. Therefore, a great effort has been made to develop analogs of vitamin D with similar antipsoriatic but diminished side effects. In the presented study the vitamin D analogs ZK203278, ZK159222 and ZK191784 had similar effects on AMP expression as calcipotriol [39]–[43]. All analogs tested induced cathelicidin expression via the VDR and involved the MEK/ERK signaling pathway similar to active 1,25D3. This effect could be exploited in other skin diseases such as atopic dermatitis (AD) where cathelicidin AMP induction is impaired upon skin infection [6], [44]. Recently it was shown that orally administered vitamin D enhances cathelicidin production in AD patients [45]. Treatment with low calcemic vitamin D analogs could therefore be used to prevent microbial superinfections in these patients. However, larger clinical trials addressing this question are needed to evaluate the efficiency and tolerability of these compounds.

In conclusion, vitamin D analogs differentially change AMP expression in keratinocytes in lesional psoriatic skin. As AMPs act as proinflammatory “alarmins” and play a role in psoriasis pathogenesis targeting their expression might be beneficial in this disease. The development of vitamin D analogs with well directed effects on AMP expression in psoriasis, but also other cutaneous diseases, might lead to new treatments for those chronic inflammatory skin diseases.

## Materials and Methods

### Patients and skin samples

All treatments and sample acquisitions, including skin biopsies, were approved by the committee on investigations involving human subjects at the Faculty of Medicine, University of Munich, Germany. For all procedures, informed written consent was obtained. Patients did not receive topical treatment before entering the study. 4-mm punch biopsies were taken from a marker psoriatic plaque before treatment with a calcipotriol containing ointment (0.005%; applied twice daily) and 5 to 7 days after treatment (n = 8). Skin biopsies from healthy (non-psoriatic) volunteers (n = 7) served as controls. Additionally, biopsies from untreated lesional and non-lesional skin from psoriasis patients (n = 6) were collected. All biopsies were directly transferred to 1 ml Trizol^®^ (Invitrogen) or cut and one half transferred to 1 ml Trizol^®^ and the other half to 200 µl RIPA-buffer (10 mM Tris-Cl, 1 mM EDTA, 1% Triton X-100, 0.1% sodium deoxycholate, 0.1% SDS, 140 mM NaCl, 1 mM PMSF). All samples were homogenized and mRNA or total protein extraction was performed and analyzed as described below.

### Cell culture and stimuli

Normal human epidermal keratinocytes (NHEK) were grown in EpiLife^®^ cell culture medium (Cascade Biologics) containing 0.06 mM calcium and 1 x EpiLife^®^ defined growth supplement (EDGS) at 37°C under standard tissue culture conditions. Stock cultures were maintained for up to six passages in this medium with the addition of 10 µg/ml gentamicin and 0.25 µg/ml amphotericin B. HaCaT keratinocytes were cultured in Dulbecco's modified Eagle's medium (DMEM) with 4.5 g/l glucose supplemented with 10% fetal bovine serum, 50 U/ml penicillin and 50 µg/ml streptomycin (PAA Laboratories). Cells at 40–60% confluence were stimulated for different time periods with 1,25-dihydroxyvitamin D3 (1,25D3) (10^−8^–10^−7^ M; Sigma), calcipotriol (10^−10^–10^−7^ M; LEO Pharma), ZK159222 (10^−10^–10^−7^ M; Bayer Schering), ZK191784 (10^−10^–10^−7^ M; Bayer Schering), ZK203278 (10^−10^–10^−7^ M; Bayer Schering) and/or IL-17A (10 ng/ml; R&D). For inhibition of MEK/ERK the MEK1 inhibitor PD98059 (20 µM; Cell Signaling) was added 1 h before stimulation.

### Quantitative real-time PCR (qPCR)

Total RNA was extracted from stimulated cells or skin samples using Trizol^®^ and approximately 1 µg RNA was reverse transcribed using DyNAmo™ cDNA Synthesis Kit (Finnzymes) according to the manufacturer's instructions. The expression of cathelicidin, HBD2, HBD3, psoriasin, IL-17A, IL-17F, IL-8, VDR, ERK1 and ERK2 was evaluated using a LightCycler^®^ 2.0 system and the corresponding human Universal Probe Library Set (Roche). The primers were designed by an algorithm on www.universalprobelibrary.com and porphobilinogen deaminase (PBGD) was used as housekeeping gene in a duplex qPCR reaction. PBGD was chosen because PBDG, cathelicidin and HBD2 belong to a low-abundance class of mRNAs and expression levels in untreated keratinocytes are low. Preliminary tests verified that expression of PBGD was not affected upon treatment of NHEK and HaCaT keratinocytes. Analyzed genes and corresponding primers are listed in Table 1. All analyses were performed in triplicate from two to three independent cell stimulation experiments. Fold induction relative to the vehicle treated control was calculated as previously described [21]. Results were considered significant when at least a two-fold difference in expression levels was detected and statistical analysis revealed *P*<0.05.

**Table 1:** Target genes and corresponding primers for qPCR.

Target Gene	Forward	Reverse
cathelicidin	5′ tcggatgctaacctctaccg 3′	5′ acaggctttggcgtgtct 3′
HBD2	5′ tcagccatgagggtcttgta 3′	5′ ggatcgcctataccaccaaa 3′
HBD3	5′ tgtttgctttgctcttcctg 3′	5′cgcctctgactctgcaataa 3′
psoriasin	5′ aagcctgctgacgatgatg 3′	5′cgaggtaatttgtgcccttt 3′
IL-17A	5′ tgggaagacctcattggtgt 3′	5′ ggatttcgtgggattgtgat 3′
IL-17F	5′ ggcatcatcaatgaaaacca 3′	5′ tggggtcccaagtgacag 3′
IL-8	5′ agacagcagagcacacaagc 3′	5′ atggttccttccggtggt 3′
VDR	5′ cttctctggggactcctcct 3′	5′ tggacgagtccatcatgtct 3′
ERK1	5′ ccctagcccagacagacatc 3′	5′ gcacagtgtccattttctaacagt 3′
ERK2	5′ ccgtgacctcaagccttc 3′	5′ gccaggccaaagtcacag 3′

### Western blot

NHEK were stimulated with 1,25D3 (10^−8^ M), calcipotriol (10^−8^ M), ZK159222 (10^−8^ M), ZK191784 (10^−8^ M), ZK203278 (10^−8^ M) and IL-17A (10 ng/ml) for up to 24 h and lysed in RIPA-buffer. Total protein was measured with the BCA^™^ Protein Assay Kit (Pierce). Equivalent amounts were separated on a NuPAGE^®^ 10% Bis-Tris Gel (Invitrogen). After separation, proteins were blotted onto an Invitrolon^™^ PVDF membrane (Invitrogen) and blocked in Tris-buffered saline (TBS) diluted 1% Western Blocking Reagent (Invitrogen) for 1 h at room temperature. Membranes were stained with antibodies detecting hCAP18/LL-37 (Innovagen), HBD2 (R&D), VDR (R&D), phospho-p44/p42 MAP Kinase (Cell Signaling), phospho-IκB-α (Ser32/36) (Cell Signaling), IκB-α (Cell Signaling), α-tubulin (Biozol) or β-actin (Sigma) overnight and reprobed with respective HRP-conjugated secondary antibodies (Dako). Stained protein was visualized using the Amersham ECL Plus^™^ Western blotting Detection System (GE Healthcare). For analyses of p44/p42 phosphorylation cells were incubated without addition of EDGS for 24 h prior to stimulation.

### RNAi and transfection

NHEK at about 30% confluence were transfected twice (48 h apart) with siRNA oligonucleotides using Lipofectamine^™^ RNAiMAX Reagent (Invitrogen) to maximize the silencing effect. Cells were transfected with either a siRNA oligonucleotide against VDR (20 nM), a mix of siRNA oligonucleotides against ERK1 and ERK2 (all at 20 nM) or a nontargeted control siRNA oligonucleotide (20 nM or 40 nM) and maintained at 37°C under standard tissue culture conditions. 4 h after the second transfection cells were stimulated with 1,25D3 (10^−8^ M), calcipotriol (10^−8^ M), ZK191784 (10^−8^ M) and ZK203278 (10^−8^ M). Keratinocytes were then harvested and evaluated by qPCR or Western blot.

### Reporter gene analysis

To analyze NF-κB promoter activity after stimulation with IL-17A, calcipotriol and 1,25D3, a fragment of the NF-κB promoter containing six NF-κB binding sites was cloned into a luciferase reporter plasmid (pGL3; Promega) and transfected into HaCaT keratinocytes. To analyze cathelicidin promoter activity after stimulation with 1,25D3 and its analogs, a 5 kb fragment of the 5′ untranslated region (UTR) of the human cathelicidin gene CAMP was cloned into a luciferase reporter plasmid (pGL4.10; Promega) and transfected into HaCaT keratinocytes. The 5 kb fragment was amplified with sense 5′ AGAGTCCGAGCTCACAACCTGGAGAGGCTGAGTCTG 3′ and antisense 5′ AGAGTCCCTCGAGGGTCCCCATGTCTGCCTCCCTCT 3′ primers using human genomic DNA as a template. Primers were designed to introduce an XhoI restriction site at the 5′ end and a HindIII restriction site at the 3′ end of the amplicon. The amplification products were digested with XhoI and HindIII and cloned into the pGL4.10 luciferase reporter vector.

The plasmids were transformed into Escherichia coli DH5α competent cells, following the manufacturer's guidelines. After DNA purification using the Qiagen Midi Plasmid Kit (Qiagen) and verification of the inserted 5 kb fragment by sequencing (Eurofins MWG Operon), HaCaT cells were transfected with the indicated reporter plasmid and the empty pGL4.74 renilla control vector (Promega). For analysis of the NF-κB promoter activity cells were stimulated 24 h after transfection with IL-17A (10 ng/ml) in the presence or absence of calcipotriol or 1,25D3 (all at 10^−8^ M), and harvested 6 h after stimulation. For analysis of cathelicidin promoter activity cells were stimulated 4 h after transfection with 1,25D3 (10^−7^ M), calcipotriol (10^−7^), ZK159222 (10^−7^ M), ZK191784 (10^−7^ M) and ZK203278 (10^−7^ M) for 24 h. Firefly luciferase activity from the NF-κB pGL3 reporter vector, the CAMP pGL4.10 reporter vector and renilla luciferase activity from the pGL4.74 control vector were measured by the Dual-Glo Luciferase Assay system (Promega) in a luminometer according to the manufacturer's instructions. Promoter activity was reported as the ratio between pGL3 or pGL4.10 and pGL4.74 activities in each sample.

### Immunofluorescence staining

Immunofluorescence stainings were performed to detect cathelicidin and HBD2 in skin from psoriasis patients before and after treatment with calcipotriol. Frozen sections of lesional skin were incubated for 1 h with antibodies detecting hCAP18/LL-37 (Innovagen) and HBD2 (R&D). The sections were then incubated with NorthernLights™ fluorescent secondary antibodies (R&D). Sections were overlayed with ProLong Gold antifade reagent with DAPI (Invitrogen) and analyzed with a TissueFAXS System microscope at 100–200× magnification and the corresponding HistoQuest software (TissueGnostics).

### Statistical analysis

All statistical analyses were performed using GraphPad Prism 4.0 (GraphPad Software Inc.). Student's *t* test or Mann-Whitney test was used to calculate statistical differences. Analysis of biopsies from lesional vs. non-lesional psoriatic skin was performed with Wilcoxon matched pairs test. Values of *P*<0.05 were considered significant and all data are displayed as means±SD.

## Supporting Information

Figure S1 Expression of HBD2 and cathelicidin in psoriatic plaques before and after treatment with calcipotriol. Immunofluorescence stainings of tissue sections from a representative patient demonstrate epithelial localisation of HBD2 and cathelicidin antimicrobial peptide before and after topical treatment with calcipotriol in lesional psoriatic skin.
(0.37 MB TIF)
