# Supplementary figures and images for: Vitamin D Analogs Differentially Control Antimicrobial Peptide/“Alarmin”Expression in Psoriasis
Source: PLoS One. 2009 Jul 22;4(7):e6340. doi: 10.1371/journal.pone.0006340 (PMC2709447; doi:10.1371/journal.pone.0006340)

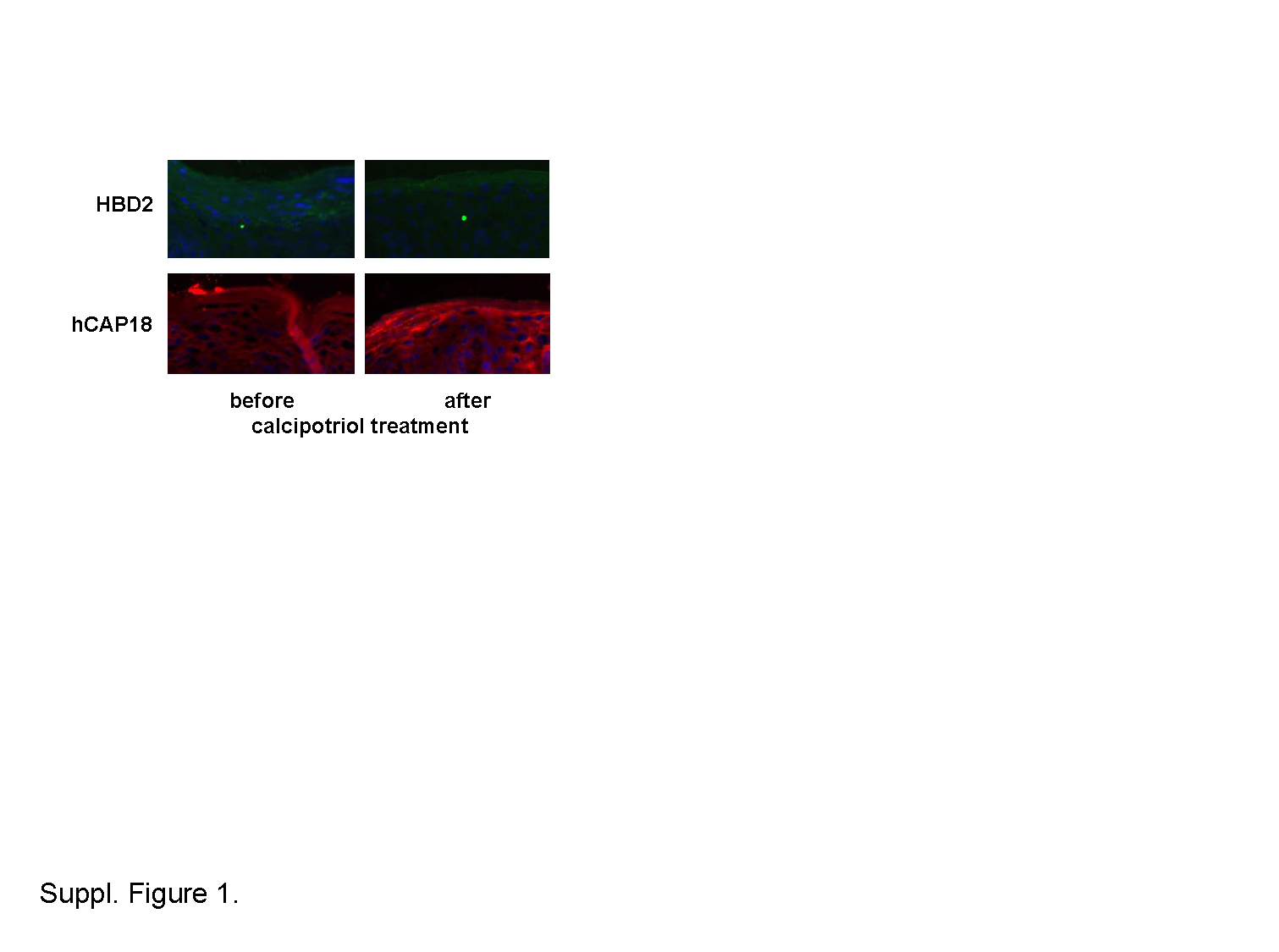

Supplement: Figure S1 — Expression of HBD2 and cathelicidin in psoriatic plaques before and after treatment with calcipotriol. Immunofluorescence stainings of tissue sections from a representative patient demonstrate epithelial localisation of HBD2 and cathelicidin antimicrobial peptide before and after topical treatment with calcipotriol in lesional psoriatic skin. (0.37 MB TIF) [file pone.0006340.s001.tif]
